# Exploration of Zero-Valent Iron Stabilized Calcium–Silicate–Alginate Beads’ Catalytic Activity and Stability for Perchlorate Degradation

**DOI:** 10.3390/ma15093340

**Published:** 2022-05-06

**Authors:** Yu-Kyung Jung, Alam Venugopal Narendra Kumar, Byong-Hun Jeon, Eun Young Kim, Taewoo Yum, Ki-Jung Paeng

**Affiliations:** 1 Department of Chemistry, Yonsei University, Wonju 26493, Korea; fts135@yonsei.ac.kr (Y.-K.J.); ssatang@yonsei.ac.kr (E.Y.K.); t7630@hanmail.net (T.Y.); 2 Department of Earth Resources and Environmental Engineering, Hanyang University, Seoul 04763, Korea; avnaren@hanyang.ac.kr

**Keywords:** perchlorate contamination, nZVI, perchlorate degradation, silicate–alginate beads

## Abstract

Perchlorate contamination in groundwater poses a serious threat to human health, owing to its interference with thyroid function. The high solubility and poor adsorption of perchlorate ions make perchlorate degradation a necessary technology in groundwater contaminant removal. Here, we demonstrate the perchlorate degradation by employing nano zero-valent iron (nZVI) embedded in biocompatible silica alginate hybrid beads fabricated using calcium chloride (1 wt%) as a crosslinker. The concentration of precursors (sodium alginate, sodium silicate) for bead formation was standardized by evaluating the thermal stability of beads prepared at different sodium silicate and alginate concentrations. Thermal degradation of silica alginate hybrid samples showed a stepwise weight loss during the thermal sweep, indicating different types of reactions that occur during the degradation process. The formation of the silica alginate hybrid structure was confirmed by FT-IR spectroscopy. Scanning electron microscopy (SEM) data revealed the surface morphology of silica alginate hybrid changes by varying sodium silicate and alginate concentrations. nZVI-loaded alginate–silicate polymer bead (nZVI-ASB) exhibited excellent perchlorate degradation efficiency by degrading 20 ppm of perchlorate within 4 h. Our study also showed the perchlorate degradation efficiency of nZVI-ASB is maximum at neutral pH conditions.

## 1. Introduction

Concerns about perchlorate (ClO_4_^−^) contamination in groundwater are increasing owing to their serious health effects on human consumption. A major portion of this contamination occurs through anthropogenic activity from the military (used as missile oxidizers) and industrial (e.g., electronic cleaning, automotive airbags, paints, medicines, fertilizer manufacturing) sources. High solubility and stability of perchlorate ions in water lead to effortless contamination in aquifers. A poor water management system could lead to perchlorate intake by humans, which inhibits iodine absorption and thyroid hormone production. Therefore, the development of perchlorate degradation methods has aroused significant interest [1,2,3].

Recently, nanotechnology has been actively applied to the treatment of environmental pollutants as it shows excellent processing capacity for many pollutants. Nanoscale zero-valent iron (nZVI) has drawn attention due to its high removal efficiency and applicability among nanoparticles used to process pollutants [4,5]. A number of studies have also reported on the application of nZVI to perchloric acid ion decomposition [6]. It is reported that reducing the size of nZVI or increasing the surface area can improve the degradation activity, resulting in enhanced reaction rates [7]. The main challenge here is managing these active nZVI particles from aggregation to maintain their activity during the treatment procedure. nZVI particles can be fixed to starch biomolecules such as alginic acid, etc., which can prevent agglomeration, thereby reducing the size of the particles and eliminating the coagulation tendency. The investigation of inorganic–organic hybrid materials has gained widespread attention in recent years [8,9,10,11,12,13,14]. Calcium–alginate hydrogel beads are one of the most commonly used carriers in the entrapment immobilization of biocatalyst owing to their significant advantages such as good biocompatibility, low cost, easy availability, and simplicity of preparation. However, some disadvantages are often associated with this carrier, including high biomolecule leakage, low mechanical strength, and serious swelling due to their open structure, large pore size, and high hydrophilicity [15,16,17,18].

To circumvent the later limitations, various strategies have been adopted, including covalent crosslinking with polymers, such as chitosan, poly(acrylic acid), and coating the surface of alginate gel beads with other reagents, such as poly-L-lysine and glutaraldehyde [19,20,21]. However, these methods often suffer from low efficiency and complexity [22]. Coradin et al. prepared a silica–alginate hybrid gel by impregnation of mesoporous silica particles with alginic acid solution and encapsulated β-galactosidase within it. The enzyme leaching was only 15% in the composite compared with 40% in Ca–alginate [8]. Compared with the pure calcium alginate gel, the hybrid gel exhibited better stability upon aging and effectively limited enzyme leaching. The diffusion coefficient (D) of glucose decreased by 10% after silica coating due to the small pore size (~20–500 Å). The association of alginate capsules with biocompatible silica species brings great opportunities and interests as it allows the combination of a soft biocompatible component (viz. alginate) with a resistant and non-swelling component (silica) [23].

In the present work, we tested the utilization of this biocompatible alginate–silicate polymer to embed nZVI using a Ca^2+^ crosslinker. By controlling the polymer solution flow rate, we were able to prepare nZVI-alginate silicate beads (nZVI-ASB) with a size <1 mm. The composition of precursors for alginate–silicate bead formation was fixed by characterizing the thermal stability of final crosslinked polymers. As-synthesized nZVI alginate–silicate polymer bead was applied for perchlorate decomposition at 90 °C. 

## 2. Materials and Methods

### 2.1. Materials Used 

Sodium alginate and sodium silicate were purchased from Aldrich chemicals. Calcium chloride was supplied from Aldrich chemicals. Sodium borohydride (NaBH_4_), a reducing agent for reducing Fe ions fixed on beads, used the products from Duksan Chemical (Korea). Acetone and sulfuric acid (H_2_SO_4_ > 96.4%) were procured from J.T. Baker (Phillipsburg, NJ, USA). Sodium perchlorate (NaClO_4_ > 98%) used as a standard substance was from Sigma-Aldrich (St. Louis, MI, USA). Sodium hydroxide (NaOH > 97%), a mobile solvent for ion chromatography, from Sigma-Aldrich (St. Louis, MI, USA), was used to measure the degradation efficiency of perchlorate ions.

### 2.2. Fabrication of Alginate–Silicate Hybrid Polymer

To prepare the hybrid alginate beads, a solution of sodium alginate (1 wt%) was mixed with different concentrations of sodium silicate solution (0.5, 1.0, 1.5, and 2 wt%). Solutions of sodium alginate and sodium silicate were prepared using de-ionized water. The solution mixture of sodium alginate and sodium silicate was added dropwise into a fixed concentration of calcium chloride (1 wt%) solution. The hybrid beads formed rapidly. The beads were collected using filtration and rinsed with distilled water several times. After that, the hybrid beads were air-dried. The detailed composition of silica alginate hybrid beads were given in Table 1.

### 2.3. Characterization of nZVI-ASB

First, the surface morphology of ASB was investigated using an SEM instrument (Hitachi, S-4100) after being sputter-coated with gold. The thermal stability of the beads was evaluated by thermogravimetric analysis (TGA) (Universal V4.1D instrument) under a nitrogen atmosphere in the temperature ranges from 18 to 1000 °C at a heating rate of 10 °C/min. The weight loss of ASB after air-drying was found to be 90–98%, and it was calculated using the formula:

% Weight loss = [(W_1_ − W_2_)/W_1_ * 100].

where W_1_ and W_2_ are the weight of the bead before and after drying, respectively. The weight loss percentage after air-drying decreases with an increase in sodium alginate concentration at a fixed concentration of sodium silicate and calcium chloride respectively, see Table 1.

The total intrusion volume, porosity, and apparent densities were measured using auto pore IV 9500 V1.05 (Micromeritics Instrument Corporation, Norcross, GA, USA) porosimeter. Mercury filling pressure (1.33 psi) and equilibration time (10 s) was used in this study. The functional groups in the ASB polymer were analyzed using Fourier transform infrared spectroscopy (FT-IR) (Perkin-Elmer Spectrum One B). The crosslinking of polymers was studied via solid-state Si NMR (Bruker spectrometer (DSX 400 MHz) at 9.4 T. The optical images of the beads were captured using a digital camera at a fixed length. An atomic absorption spectrometer (AAS) was used to determine the iron content in nZVI-ASB. To confirm the degree of reductive decomposition of perchlorate ions, ion chromatography (IC) was employed.

### 2.4. Fabrication of nZVI Loaded Alginate–Silicate Polymer Bead (nZVI-ASB) 

**Scheme 1:**
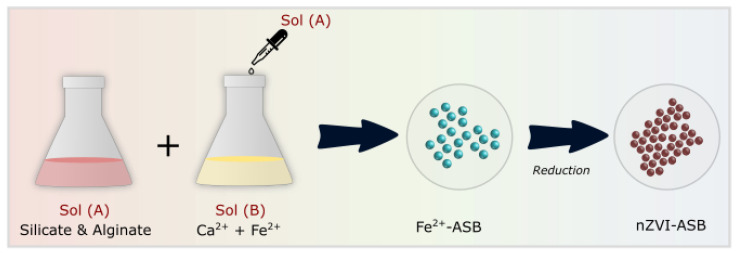
Schematic diagram showing the steps involved in the formation of nZVI-ASB.

The overall process of nZVI-ASB synthesis is shown in Scheme 1. Briefly, to produce a stable ZVI, the iron ion (Fe^2+^) must be fixed in the alginate’s polymer chains of the manufacturing circular beads. However, using only metal ions does not produce circular beads. Calcium ions (Ca^2+^), which are more binding to alginates than iron ions, can effectively hold iron ions within polymer beads. Therefore, it produces a very stable circular bead. Fe–Alginate–Silicate (nZVI-ASB) hybrid polymers were prepared to reduce iron ion ejection during reduction and prevent degradation of polymer frames due to iron ion escape during the perchloric acid ion decomposition. Two solution mixtures were prepared separately for the fabrication of nZVI-ASB. Initially, 2% FeCl_2_ and CaCl_2_ solution (100 mL) was prepared. Then, a 1 wt% sodium alginate solution and 1.5 wt% sodium silicate solution were prepared. Both solutions were stirred at a speed of 200 rpm for 2 h. Then, using a peristaltic pump (RAININ), 50 mL of Na–alginate–silicate solution was pumped slowly into a FeCl_2_+CaCl_2_ mixture through 7 mm diameter silicon tubing connected with a syringe at the end. Afterward, the mixture was stirred for 2 h to produce Fe^2+^-loaded alginate–silicate beads. The beads were collected using filtration and rinsed with distilled water several times. After that, the hybrid beads were dried in an oven at 60 °C for more than 48 h to remove moisture contained in the beads, which was followed by the addition of an excess of a 0.5 M sodium borohydride (NaBH_4_) solution after which it reacted for more than 12 h. Later, a reaction step was carried out in a nitrogen chamber to avoid Fe oxide formation. During the reduction reaction, hydrogen gas bubbles were generated, and the color of the beads changed completely. To wash the beads after the reaction, nitrogen was introduced for 10 min, and then the beads were washed five or more times with deionized water (DI) and acetone and then dried for more than 24 h. For a precursor optimization study, the solution mixture of sodium alginate and sodium silicate was added dropwise into a fixed concentration of calcium chloride (1 wt%) solution. The characterization of these samples was tested after several washing and drying steps. Finally, the reloading of used beads was performed at room temperature, by mixing 100 mL of 2% FeSO_4_ 6H_2_O solution in a 200 mL vial and stirring for 24 h.

### 2.5. Perchlorate Degradation Using nZVI-ASB 

The perchlorate degradation experiments were carried out in batches. Perchlorate reaction solution was prepared as a 20 μg/mL solution and stirred for 10 min. For this solution, a nitrogen gas was introduced for 20 min to eliminate the interference of dissolved oxygen. In order to maintain the pH of the reaction solution at the highest decomposition efficiency (pH 7), a potassium dihydrogen phosphate buffer solution was used. Exactly 50 mL of perchlorate solution and 1 g of reduced nZVI-ASB were taken in a round bottom flask, and then the reduction reaction was carried out at 90 °C in a heating mantle. In order to confirm the decomposition efficiency of perchlorate ions by time, 200 μL of the sample from the reaction vessel was taken every hour and filtered with a cellulose acetate syringe filter (0.45 μm: ADVANTEC) and analyzed by ion chromatography.

## 3. Results and Discussion

### 3.1. Characterization of Alginate/Silicate Hybrid Polymer

**Figure 1:**
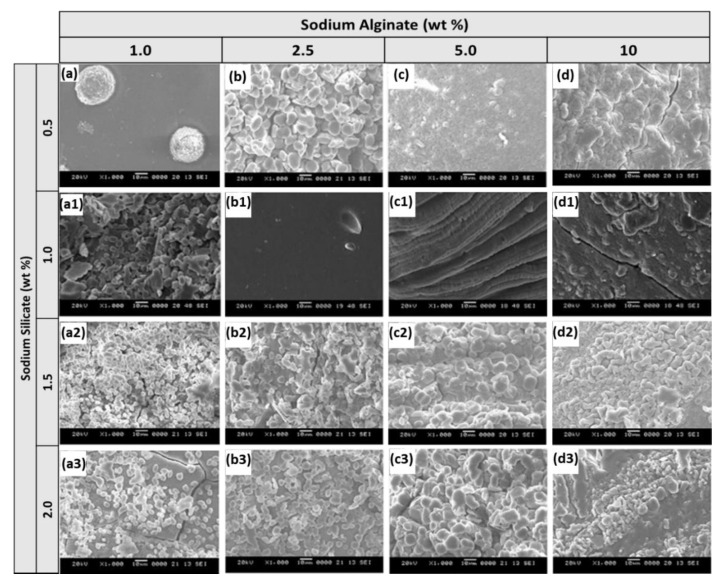
SEM images of ASB synthesized by fixing 0.5 (**a**–**d**), 1.0 (**a1**–**d1**), 1.5 (**a2**–**d2**) and 2.0 (**a3**–**d3**) wt% of sodium silicate and varying sodium alginate precursor from 1 to 10 wt% (scale 10 μm).

First, we were interested in evaluating the physical properties of our hybrid polymer prepared by varying the precursor concentrations since the physicochemical properties directly affect the characteristics of a hybrid material [24]. The surface morphology of these samples was first examined using SEM. Variations in the surface characteristics at different wt% of alginate and silicate are shown in Figure 1. First, the alginate concentration was fixed to 1, 2.5, 5, and 10 wt% in the reaction vial and increased the silicate concentration from 0.5 to 2.0 wt% with increments of 0.5 wt%. At low silicate precursor concentration, the surface of the crosslinked polymer exhibited a very dense matrix with some small pores and cracks. Upon increasing the silicate in the reaction vial, a large amount of small size particles was observed. The particles formed had uniform sizes and were distributed evenly on the surface. At higher alginate concentrations, the particles started to aggregate and dominate the surface with silica particles. From the SEM data, we found that at higher silicate concentrations, the roughness of the polymer surface increased. As a result, we deduced it would benefit from having a high surface area. 

**Figure 2:**
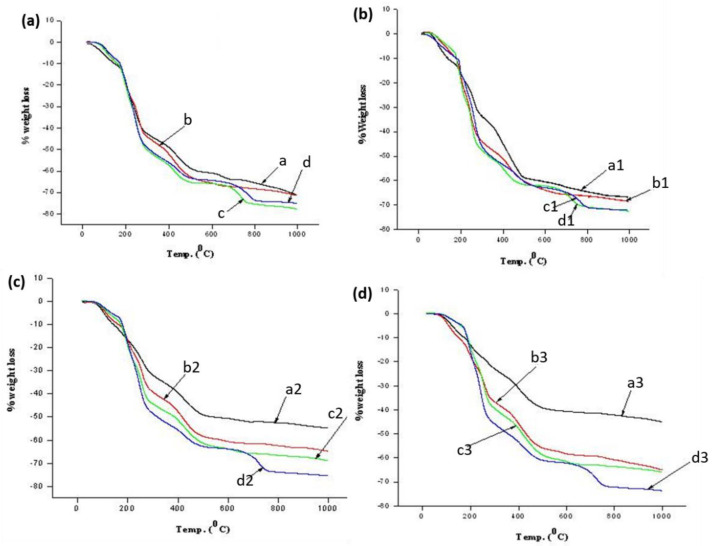
TG profiles of ASB prepared at fixed concentrations of sodium alginate 0.5 (**a**), 1.0 (**b**), 1.5 (**c**) and 2.0 (**d**) wt% and varying sodium silicate concentrations.

To evaluate the exact variation in the degradation trend of ASB with change in the sodium alginate and sodium silicate concentrations, thermogravimetric analysis was carried out. We calculated the weight loss of the beads at various temperatures from the TGA profiles (see Figure 2a–d), which are presented in Table 2. The weight loss in ASB increased with an increase in sodium alginate concentration from 1 to 5 wt% at 0.5 wt% of silicate. Then, a sudden decrease of 10 wt% of sodium alginates was demonstrated. This can be explained by the concentration of free surface functional groups. There was greater weight loss with an increase in sodium alginate concentrations due to the higher concentration of free functional groups in alginate molecules, whereas with 10 wt% of sodium alginates, the weight loss percentage was lower compared to 5 wt% of sodium alginate, due to effective crosslinking between two polymers at this concentration. Similarly, we noticed that the weight loss increased with an increase in sodium alginate concentration from 1 to 5 wt% with silicate concentration of 1 wt%; again, a slight decrease at 10 wt% was observed a at fixed concentration of calcium chloride (1 wt%). This is again attributed to the reason stated above for samples in Figure 1a–c. However, the decrease in weight loss trend was not found when using higher silicate concentrations (i.e., above 1 wt%). In addition, the results revealed that the ASB sample prepared at a high concentration of sodium silicate was more stable at high temperature (1000 °C) compared to a low concentration of sodium silicate. Among these compositions, sample a3 demonstrated the highest thermal stability, which is due to the effective crosslinking between inorganic and organic materials. 

Desimone et al. reported that the optimization of pore size and morphology must be addressed to understand the nature of these inherently complicated materials and the mechanism of their interaction, because it will determine the resulting function performance [14,25]. Therefore, the effect of sodium silicate and alginate concentrations on porosity, surface morphology, as well as thermal degradation of ASB was investigated. Before drying, the surface of the ASB was smooth with a spherical shape whereas the dried ASB exhibited a rough surface. Polona et al. reported a similar observation for alginate beads, which they found to be a key determinant of uniform bead formation [26]. The prepared ASB was carefully analyzed to determine their porosity which is a key parameter that governs both diffusions of chemical species and stability of the anchoring species. The total intrusion volume and porosity of ASB upon varying sodium alginate concentrations (1, 2.5, 5 and 10 wt%) at fixed concentrations of sodium silicate (0.5 wt%) and calcium chloride (1 wt%) are 0.05, 0.03, 0.01, and 0.01 mL/g and 11.95, 5.09, 3.09, and 3.43, respectively (Table 3). Both porosity and the total intrusion volume decreased with an increase in sodium alginate concentrations from 1 wt% to 5 wt% at fixed concentrations of sodium silicate (0.5 wt%) and calcium chloride (1 wt%). This is due to the more densely packed arrangement of alginate molecules at high sodium alginate concentrations. ASB formed in various other compositions were also analyzed, and it was found that the highest porosity was obtained for the a2 composition. The density of the ASB is also an important factor to consider for the structural stability of the beads during practical application.

**Figure 3:**
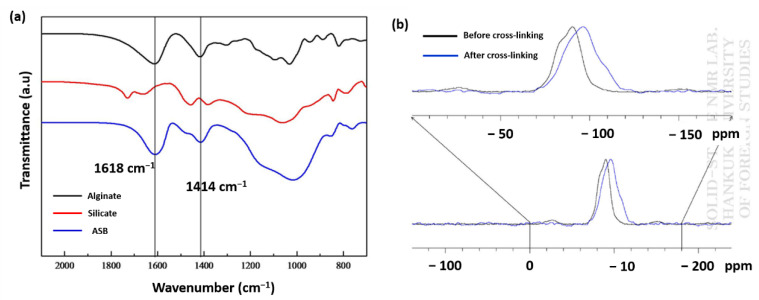
FTIR spectra of alginate and silicate and ASB (**a**). Solid-state NMR spectrum of silicate and ASB (**b**).

The functional groups present in the prepared beads were investigated using FTIR spectroscopy. FTIR spectra of sodium silicate, simple calcium alginate, and silica alginate hybrid samples are depicted in Figure 3a. The bands appeared at 1618 and 1414 cm^−1^ in both calcium alginate, and the hybrid samples and were assigned to asymmetric and symmetric stretching peaks of the –COO^−^ group of calcium alginate, suggesting the crosslinking process had less interference on the carboxyl functional group of alginates. The peaks around 1030 cm^−1^ were attributed to the stretching of the C-O-C group of calcium alginate; the same observation was also reported in other studies [27,28,29,30]. In the case of hybrid beads, the band that appeared at 1083 cm^−1^ was broadened, which is due to symmetric stretching of Si-O-Si groups in addition to the C-O-C in the polymer structure [31]. 

The degree of polymerization of silicate after crosslinking two precursors was also studied by conducting ^29^Si solid-state NMR experiments for sodium silicate and ASB [32]. We found the signals of sodium silicate at −90 ppm, which shifted to −96 ppm after crosslinking with alginate. The chemical shift values displayed in Figure 3b clearly indicate that there was a change in the coordination of the Si atoms upon crosslinking. It also provides details about the number of Si-O-Si bonds through Q^n^. From the NMR signal, the n value for the crosslinked sample was found to be 3.0, suggesting a single Si atom in ASB is bonded to three Si atoms through oxygen is represented by Q^3^ [33] and effective crosslinking between inorganic silica during ASB formation. Previously, our TGA results clearly indicated samples a2 and a3 as the most stable precursor composition for the preparation of ASB. Their bulk density and the porosity values were also higher than other studied compositions. In fact, the porosity of ASB formed using the a2 (18%) composition is almost double that of the a3 (8.94 %) sample. Based on these results, we choose a2 precursor composition for zero-valent Fe incorporated ASB (nZVI-ASB).

### 3.2. Investigating the Decomposition Conditions of Perchloric Acid Using nZVI-ASB 

**Figure 4:**
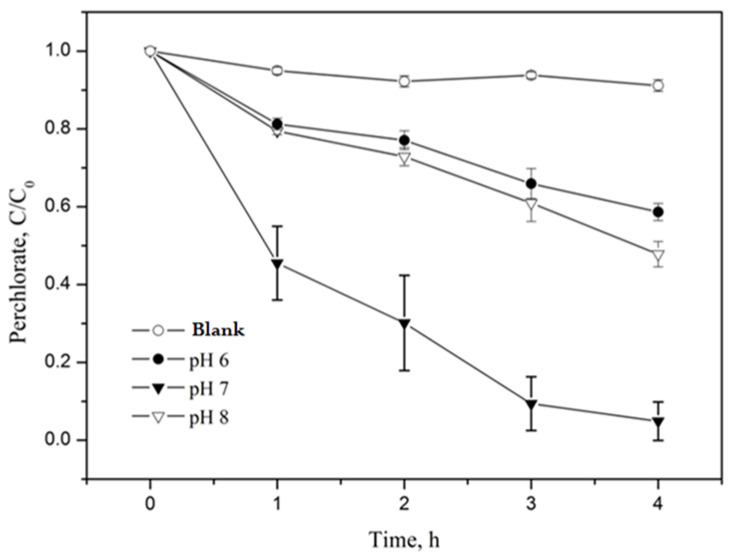
Decomposition efficiency of perchlorate by nZVI-ASB (1 g), perchlorate (20 μg/mL) and pH = 6, 7 and 8, @90 °C.

Since the hybrid catalytic material prepared in this study was composed of Fe particles, its reactivity in the aqueous phase depended on H^+^ concentrations [34]. Subsequently, the perchlorate degradation by nZVI-ASB greatly relies on the pH of the solution. Therefore, we first evaluated the perchlorate decomposition trend with nZVI-ASB in both acidic and alkaline conditions (pH = 6, 7, and 8). The study was carried out by mixing 1 g of nZVI-ASB and 50 mL of 20 mg/L perchlorate ion solution with batch type at 90 °C. A phosphate buffer system was used in order to maintain the solution pH. Potassium dihydrogen phosphate (KH_2_PO_4_) was used as the buffer solution, and the experiments were conducted at pH 6, 7, and 8. Figure 4 shows the decomposition efficiency by reacting at 90 °C for 4 h, and the control test was simultaneously performed in the presence of ASB. Our results clearly revealed that the perchlorate decomposition was faster at neutral pH conditions. At pH 8, the perchlorate degradation was sluggish, and ~50% of perchlorate removal was possible even after 4 h, which was attributed to the formation of Fe hydroxides on the nZVI surface at this pH. This passivated film had a tendency to decrease the nZVI reactivity, and it was previously noticed by Xiong et al. under similar experimental conditions [35]. The degradation of perchlorate at slightly acidic (pH 6) conditions displayed the lowest degradation performance of all the pH examined in this study due to the fact that Fe corrosion is more favored than the direct degradation of perchlorate. In the neutral condition, the surface passivation by hydroxides and Fe corrosion was ruled out as it solely promoted the reaction between ASB incorporated nZVI and the perchlorate. Studies with ZVI incorporate in other biopolymers showed less of an effect on perchlorate degradation kinetics with the pH [36]. However, the present study clearly proved that the solution pH has an imperative role to play in perchlorate degradation with the nZVI-ASB system. 

### 3.3. Stability/Reusable Capabilities of nZVI-ASB

**Figure 5:**
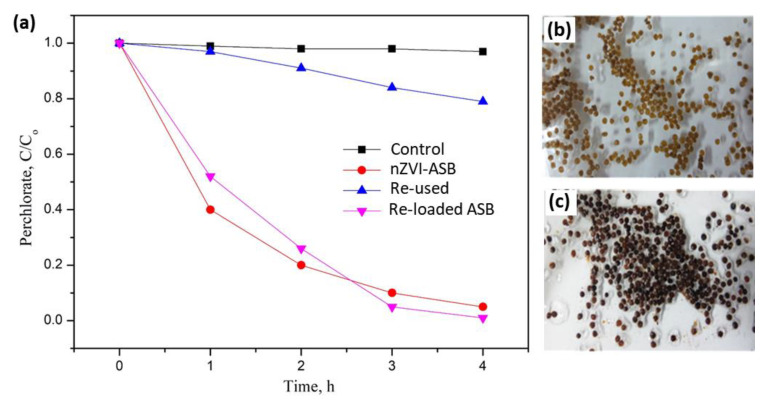
Perchlorate degradation efficiencies of pure, used and reloaded nZVI-ASB (**a**). Optical images of nZVI-ASB after perchlorate degradation (**b**) and Fe reloaded beads (**c**).

Under the optimized pH conditions, the perchlorate decomposition with 1g of nZVI-ASB and 50 mL of 20 mg/L perchlorate ion was found to show high degradation efficiency (Figure 5). To identify the reusability of nZVI-ASB, the beads used for perchlorate degradation for 4 h were recovered and washed with deionized water and acetone, and then dried. Then, the recovered beads were employed for perchlorate decomposition reaction by adopting a similar experimental condition. Our results revealed that the perchlorate degradation reduced significantly with the reused beads during the same time intervals. After 4 h of reaction, the reused beads decomposed only 25% of perchlorate with the same nZVI-ASB dosage as shown in Figure 5a. Compared with the new nZVI-ASB, the decomposition efficiency decreased by about 75% when it was reused. The decrease in the efficiency was attributed to the oxidation of ZVI in the beads during the perchlorate degradation process. This was confirmed by looking at the color of the reacted beads. It demonstrated that the ZVI incorporated in the beads was oxidized and the color of the nZVI-ASB changed dramatically from a brown color to pale yellow (Figure 5b). The lack of ZVI in the ASB matrix suppressed the perchlorate degradation in the used beads. However, the polymer matrix of ASB remained stable even after the perchlorate degradation process, indicating the high robustness of the beads which stemmed from the optimized precursor composition. Inspired by the stability of the ASB polymer matrix, we utilized these reacted beads for the incorporation of ZVI to regain its activity. For this, the reacted beads were first treated with the iron solution to anchor the Fe^2+^ species on the polymer matrix. This was followed by the reduction step to convert all the Fe^2+^ to ZVI, and obtained samples were categorized as reloaded beads. Overall, the reloaded beads resembled the pristine form of nZVI-ASB in color and textural properties (Figure 5c). The perchlorate decomposition with the reloaded beads showed more than 95% efficiency in 4 h of time. Compared with pristine nZVI-ASB, the perchlorate degradation with the reloaded beads takes place slowly during the initial hours. However, after 2 h, the reaction takes place slightly faster, and the final degradation was better than the nZVI-ASB, suggesting the multiple loading technique not only benefits the ASB utilization, but it also promotes the perchlorate degradation kinetics in a considerable proportion. This study clearly demonstrated the promising nature of the tailored ASB for the incorporation of ZVI species and perchlorate removal. The high reversibility of the bead showed the versatile nature of the hybrid polymer that helped in regaining its chemical activity. 

## 4. Conclusions

In summary, calcium–silicate–alginate hybrid polymer beads were used as a stabilizer to immobilize nZVI. The crosslinking efficiency after polymerization was studied through FT-IR and solid-state ^29^Si NMR spectra. Our study also demonstrates that the surface morphology and thermal properties of the ASB can be fine-tuned by altering the precursor ratio during the bead formation. Thermal stability studies with TGA have shown that ASBs formed with 2.0 and 1.0 wt% of silicate and alginate exhibit the highest thermal stability, respectively. As-prepared nZVI-ASB showed excellent activity for perchlorate degradation under neutral pH conditions. A reusability study showed that the perchlorate degradation efficiency of nZVI-ASB decreased due to the oxidation process of ZVI. However, after iron supplementation was performed by mixing with iron solution, the decomposition efficiency reached more than 95% in 4 h, which was similar to that of fresh nZVI-ASB. These findings suggest that ASB prepared under optimized precursor concentration could be reused through recovery and iron supplementation processes. 

## Figures and Tables

**Table 1:** Preparation of ASB by varying sodium alginate and sodium silicate concentration at a fixed concentration of calcium chloride.

Sample Name	Sodium Alginate (wt%)	Sodium Silicate (wt%)	Calcium Chloride (wt%)	wt% loss after Air Drying
a	1	0.5	1	98.05
b	2.5	0.5	1	97.45
c	5	0.5	1	95.86
d	10	0.5	1	94.12
a1	1	1	1	97.16
b1	2.5	1	1	97.28
c1	5	1	1	95.33
d1	10	1	1	94.09
a2	1	1.5	1	97.78
b2	2.5	1.5	1	96.76
c2	5	1.5	1	95.57
d2	10	1.5	1	90.40
a3	1	2	1	97.70
b3	2.5	2	1	96.76
c3	5	2	1	95.57
d3	10	2	1	90.40

**Table 2:** Thermal analysis of ASB prepared at various Silicate alginate ratios.

Sample Name	% Weight Loss of ASB at Different Temperature (°C).									
	100	200	300	400	500	600	700	800	900	1000
a	5.82	19.25	42.64	49.01	58.67	60.94	64.01	65.59	67.88	71.25
b	3.27	19.55	44.08	52.19	62.67	65.63	67.31	68.32	69.40	71.32
c	3.47	20.58	50.20	57.33	65.23	65.87	68.55	75.41	76.39	77.62
d	2.44	18.49	49.18	56.09	63.28	64.37	66.41	73.69	74.35	74.99
a1	5.59	16.28	33.57	45.08	58.84	60.49	62.71	64.66	66.15	66.69
b1	2.77	17.30	44.01	51.08	60.28	63.94	65.68	66.32	67.35	68.52
c1	2.21	19.20	47.55	55.12	61.59	62.28	65.51	70.81	71.65	72.54
d1	4.38	16.09	45.92	53.55	60.48	62.95	64.87	70.99	71.73	72.05
a2	4.25	16.81	31.95	39.66	49.07	50.62	52.02	52.51	53.58	54.76
b2	3.12	16.88	39.23	47.83	58.41	60.64	61.52	62.38	63.33	64.81
c2	2.71	19.39	44.39	51.45	61.38	63.80	65.74	66.52	67.30	68.82
d2	2.13	18.31	48.55	55.76	62.73	63.78	67.62	73.81	74.55	75.35
a3	2.34	12.89	22.89	31.11	39.20	40.61	41.35	42.14	43.39	45.01
b3	3.55	17.50	36.66	45.76	55.91	58.36	59.17	60.78	62.73	65.04
c3	0.91	13.67	39.44	47.89	58.84	61.59	62.88	63.64	64.56	65.85
d3	1.02	15.70	45.81	53.44	61.04	62.20	66.28	72.18	72.85	73.71

**Table 3:** Pore characteristic of ASB by varying sodium alginate and sodium silicate concentration at a fixed concentration of calcium chloride.

Sample Name	Total Intrusion Volume (mL/g)	Porosity (%)	Bulk Density (g/mL)	Apparent Density (g/mL)
a	0.0525	11.9591	2.2780	2.5874
b	0.0336	5.0924	1.5150	1.5963
c	0.0153	3.0963	2.0252	2.0899
d	0.0177	3.4383	1.9417	2.0109
a1	0.0903	0.3361	0.0372	0.0374
b1	0.0352	0.1481	0.0421	0.0421
c1	0.0306	0.1560	0.0510	0.0511
d1	0.0243	0.1695	0.0698	0.0700
a2	0.1081	18.0628	1.6707	2.0391
b2	0.0598	10.4051	1.7400	1.9421
c2	0.0294	5.0000	1.7023	1.7919
d2	0.0265	5.5343	2.0871	2.2093
a3	0.0499	8.9412	1.7935	1.9696
b3	0.0454	8.0920	1.7812	1.9380
c3	0.0326	5.6307	1.7264	1.8295
d3	0.0299	5.5616	1.8621	1.9718

## Data Availability

Not applicable.
